# Association between depression and stroke risk in adults: a systematic review and meta-analysis

**DOI:** 10.3389/fneur.2024.1331300

**Published:** 2024-04-25

**Authors:** Farheen Ashraf, Muhammad Saqlain Mustafa, Muhammad Ashir Shafique, Abdul Haseeb, Abdullah Mussarat, Amber Noorani, Burhanuddin Sohail Rangwala, Fatimah Kashif Rasool, Mohammad Arham Siddiq, Javed Iqbal

**Affiliations:** ^1^Department of Medicine, Jinnah Sindh Medical University, Karachi, Sindh, Pakistan; ^2^Department of Medicine, King Edward Medical University, Lahore, Punjab, Pakistan

**Keywords:** stroke-diagnosis, depression, ischemic stroke, transient ischemic attack (TIA), hemorrhagic stroke

## Abstract

**Introduction:**

Stroke is a significant global health concern, and numerous studies have established a link between depression and an increased risk of stroke. While many investigations explore this link, some overlook its long-term effects. Depression may elevate stroke risk through physiological pathways involving nervous system changes and inflammation. This systematic review and meta-analysis aimed to assess the association between depression and stroke.

**Methodology:**

We conducted a comprehensive search of electronic databases (PubMed, Embase, Scopus, and PsycINFO) from inception to 9 April 2023, following the Preferred Reporting Items for Systemic Review and Meta-analysis (PRISMA) and Meta-analysis Of Observational Studies in Epidemiology (MOOSE) guidelines. We included all articles assessing the association between different stroke types and depression, excluding post-stroke depression. Two investigators independently extracted data and assessed quality using the Newcastle–Ottawa Scale and Cochrane Risk of Bias tool, utilizing a random-effects model for data synthesis. The primary outcome was the association of depression with stroke, with a secondary focus on the association of antidepressants with stroke.

**Results:**

The initial search yielded 10,091 articles, and 44 studies were included in the meta-analysis. The pooled analysis revealed a significant association between depression and stroke risk, with an overall hazard ratio of 1.41 (95% CI 1.32, 1.50; *p* < 0.00001), indicating a moderately positive effect size. Subgroup analyses showed consistent associations with ischemic stroke (HR = 1.30, 95% CI 1.13, 1.50; *p* = 0.007), fatal stroke (HR = 1.39, 95% CI 1.24, 1.55; *p* < 0.000001), and hemorrhagic stroke (HR = 1.33, 95% CI 1.01, 1.76; *p* = 0.04). The use of antidepressants was associated with an elevated risk of stroke (HR = 1.28, 95% CI 1.05, 1.55; *p* = 0.01).

**Conclusion and relevance:**

This meta-analysis indicates that depression moderately raises the risk of stroke. Given the severe consequences of stroke in individuals with depression, early detection and intervention should be prioritized to prevent it.

**Systematic review registration:**

Prospero (CRD42023472136).

## Introduction

Stroke ranks among the primary causes of mortality and functional impairment worldwide (1). Meanwhile, depression is highly prevalent in the general population, with an estimated occurrence of 5.8% among men and 9.5% among women experiencing a major depressive event within a 12-month timeframe (2). Prior studies suggest a correlation between major depressive disorder and depressive symptoms and an increased risk of developing any form of stroke (3, 4). Numerous investigations have explored the connection between depression and stroke, with some focusing solely on the initial measurements of depression or depressive symptoms as well as potential confounding factors. However, such studies may not adequately capture the long-term implications of depression on the risk of stroke (5). Several potential physiological pathways exist by which depression may elevate the risk of stroke, including neuronal endocrine effects such as the activation of the sympathetic central nervous system and imbalances of the hypothalamic–pituitary–adrenocortical axis. Additionally, depression can lead to changes in behavior and have immunological/inflammatory effects, resulting in elevated levels of C-reactive protein (CRP), interleukin-1 (IL-1), and interleukin-6 (IL-6), all of which can contribute to the risk of stroke (6–8). The association between depressive symptoms and an increased risk of stroke in older adults has been well-documented across previous studies, although predominantly evaluated within high-income nations (3, 4, 9, 10). A single study has indicated that the prevalence of heightened depressive symptoms is higher among the Hispanic population (33%) and the Black population (27%) compared to the white population/other groups (18%). Hence, it is important to consider the influence of race/ethnicity when assessing the relationship between depression and stroke (10). Hence, we conducted a systematic review and meta-analysis to assess the relationship between depression and stroke incidence in adults.

## Materials and methods

### Search strategy

A comprehensive review of the existing literature was conducted following the guidelines outlined in the Preferred Reporting Items for Systemic Review and Meta-analysis (PRISMA) (11) and Meta-analysis Of Observational Studies in Epidemiology (MOOSE) guidelines. An extensive search was performed across multiple electronic databases, including PubMed, Embase, Scopus, and PsycINFO, covering the period from inception to 9 April 2023. The search query used was as follows: “(((((((Depression) OR (MeSH Terms: Depressive disorder)) OR (Symptoms of depression)) OR (Major depressive disorder)) AND (Stroke))) OR (Title: Risk of stroke))” to retrieve relevant articles.

### Study selection

Two investigators (MSM and MAS) independently reviewed the titles and abstracts of the identified citations. In cases where the eligibility of a study could not be determined from the abstract alone, a thorough evaluation of the full-text article was conducted. Any disagreements were resolved by a third independent investigator, FA. The articles retrieved from the systematic search were imported into the EndNote reference library, specifically version 20.2 (Clarivate Analytics), and duplicate entries were subsequently removed. The inclusion criteria consisted of Observational Cohorts and Randomized Control trials involving non-institutionalized adults aged 18 years and older. The authors of these studies reported the risk ratio or hazard ratio (HR) of stroke morbidity or mortality in individuals with depression compared to those without depression. Various forms of stroke (complete, fatal, non-fatal, ischemic, and hemorrhagic) were considered, along with different methods, for assessing depression status, such as scales or clinical diagnosis. Studies focusing on post-stroke depression were excluded. Studies focusing on cardiovascular diseases with no stroke data were excluded too. The screening process initially involved reviewing the titles and abstracts of the identified studies, followed by a comprehensive assessment of the full-text articles.

### Data extraction and quality assessment

The process of data extraction and quality assessment involved three team members, MAS, MSM, and AM, who followed established protocols. The accuracy of the data was verified by all three members, and any discrepancies were resolved through consultation with a fourth team member. Relevant information was collected from the studies, including study parameters (study name, authors, publication year, study location, duration of follow-up, and participant numbers) and participant characteristics (average age and gender distribution as male or female percentage). The primary focus was on depression, which was assessed through self-stated scales or clinician diagnosis at baseline or subsequent updates. The primary outcome of interest was the risk of stroke, with stroke types identified from self-reports, death records, or medical records. Additionally, the use of antidepressants and their association with stroke were considered during the process of data extraction and analysis. These details were included to explore potential connections between antidepressants and stroke outcomes.

### Data synthesis and analysis

The statistical analyses in this study were conducted using RevMan (Version 5.4; Copenhagen: The Nordic Cochrane Centre, The Cochrane Collaboration, 2020) (12) and Comprehensive Meta-Analysis Software Version 3.3. HRs accompanied by their corresponding 95% confidence intervals (CIs) were used as the measure of association, and a random-effects model was utilized to combine the results. Forest plots were generated to visually assess the pooled outcomes. Subgroup differences were evaluated using the chi-square test. HRs were used as the prevailing measure of association across the research studies, with relative risks (RRs) deemed analogous to HRs. When data regarding the overall occurrence of stroke were not available, data pertaining to strokes of ischemic nature, strokes resulting in death, or strokes causing non-survivable outcomes (in sequential order) were utilized as substitutes for the total number of strokes. In cases where studies presented graded relationships (such as mild, moderate, and severe depressive symptoms), only the estimates for the highest category were taken into consideration. Heterogeneity among the studies was assessed using Higgins I2, with a value below 50% deemed acceptable. Begg’s test and a visual examination of the funnel plot were conducted to evaluate publication bias. A significance level of *p* of <0.05 was considered statistically significant for all analyses.

## Results

### Literature search

**Figure 1:**
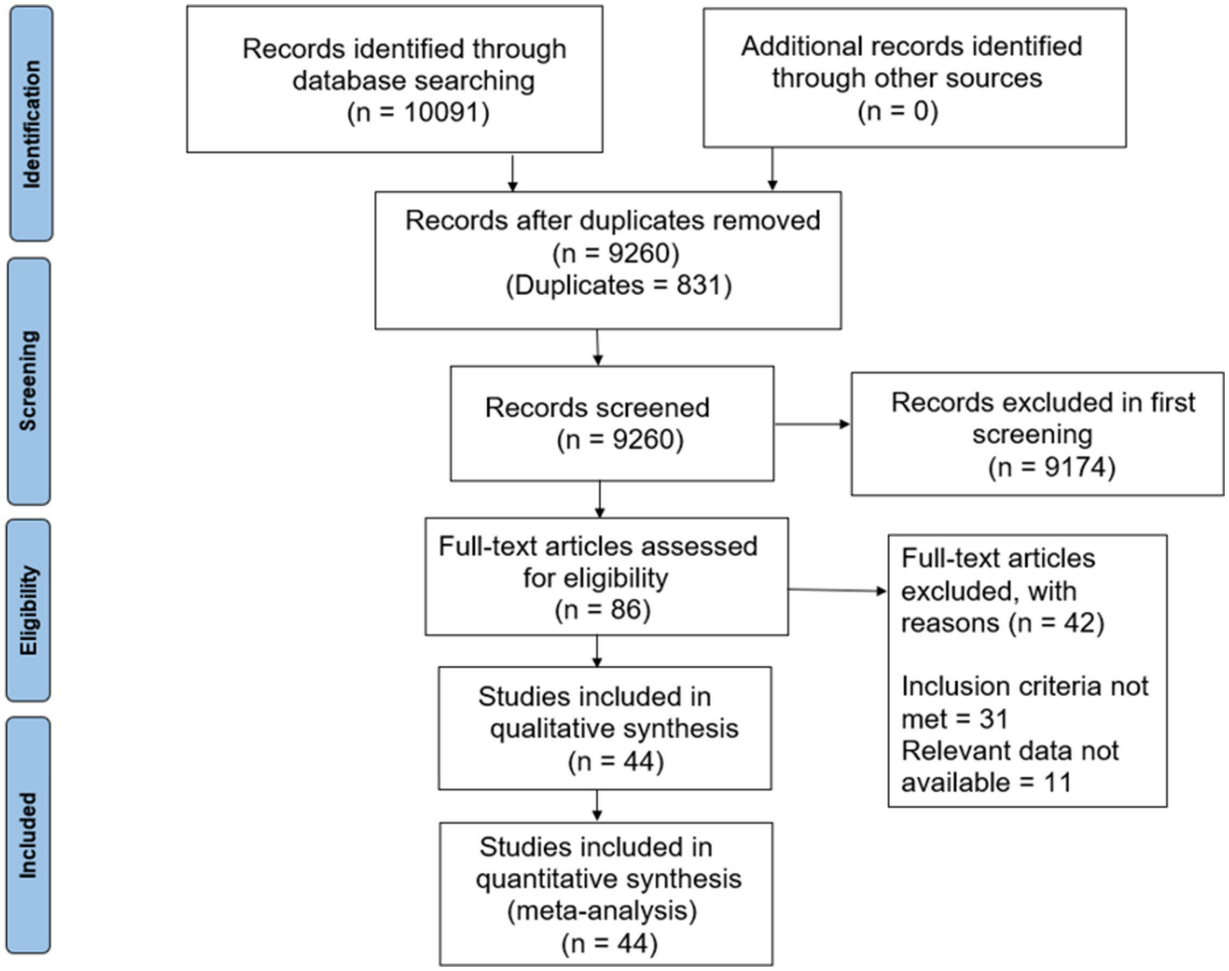
PRISMA flowchart.

The initial search yielded a total of 10,091 articles. After removing duplicates (831 records), 9,260 unique records remained for screening. All 9,260 records were evaluated based on their titles and abstracts, resulting in 86 articles for further evaluation. Following a detailed examination, 42 articles were excluded due to various reasons, including failure to meet inclusion criteria and unavailability of relevant data. Ultimately, 44 articles met the inclusion criteria and were included in the meta-analysis (Figure 1).

### Study characteristics

Among the initially identified articles, a total of 44 studies met the inclusion criteria and were included in the meta-analysis (Table 1).

**Table 1:** Baseline characteristics of included studies.

Study name	Country	No. of participants	No of cases	Mean follow-up years	Mean age (years)	Male %	Female %	Duration, y	Depression measures	Stroke measures
Gilsanz 2017 (5)	USA	4,319	334	8.52	72.36 (5.23)	41.63	58.37	2	CES-D	Death certificates, neurological images (MRI and CT scan)
Meza 2020 (13)	Mexico	10,693	546	11.4 (4.2)	62.3 (9.4)	45.15	54.84	2	CES-D	Clinical diagnosis AND medical records.
Ford 2021 (14)	USA	30,239	1,262	9.21 (4.0)	64.5	45	55	NA	CES-D	The physician committee independently reviewed medical and death records obtained after a reported or suspected stroke event.
Tsai 2017 (15)	Taiwan	40,645	4,550	12	NA	31.1	68.89	NA	Clinical diagnosis	Death certificates, medical records, and health insurance data.
Pan 2011 (16)	USA	80,574	1,033	6	66	0	100	7	MHI-5	National survey of stroke criteria, medical record, and autopsy reports.
Sun 2016 (17)	China	487,377	27,623	7.2	51	40.9	56.9	9	(DSM)-IV	Death certificates, medical records, and health insurance data.
Majed 2012 (18)	Ireland, France	9,601	136	5	54.98	100	0	10	CES-D	Follow-up and death certificates.
Qiao 2021 (19)	Europe (19 countries)	78,212	2,173	12	NA	43.1	56.38	14	(EDS)	Death certificates and follow-up data.
Seifert 2012 (20)	Germany	3,908	156	6.13	NA	41.19	58.77	3	GDS	ICD classification
Ellis 2010 (21)	USA	10,025	1,925	8	NA	43.1	56.9	5	CES-D	Death certificates
O Brien 2015 (22)	USA	5,301	738	10	NA	NA	NA	5	CES-D	Review of death certificates and abstraction of medical records.
Henderson 2013 (23)	USA	4,120	603	6	77.1 (6.3)	58.2	61.4	4	CES-D	Medical records and clinical diagnosis.
Glymour 2012 (10)	USA	18,648	1,998	3.16	NA	NA	NA	NA	CES-D	Interviews from patients and relatives.
Glymour 2010 (24)	USA	19,087	1854	7.5 (3.4)	NA	NA	NA	NA	CES-D	Medical records during follow-up and death certificates.
Freak-Poli 2018 (25)	Netherlands	8,129	1,344	15	NA	NA	NA	21	CES-D	Follow-up data of clinical diagnosis and medical records.
Li 2019 (26)	China	12,417	1,088	4	58.4 (9.51)	49.2	50.8	4	CES-D	Follow up data.
Gafarov 2013 (27)	Russia	560	35	16	NA	0	100	15	The questionnaire MOPSY (subscale D)	NA
Daskalopoulou 2016 (28)	UK	1,937,360	94,432	6.9	NA	NA	NA	13	Clinical diagnosis	Follow-up and death certificates.
Vogt 1994 (29)	USA	2,573	NA	15	NA	46.1	53.9	15	Depressive Index	Death index or vital records.
Everson 1998 (30)	USA	6,676	169	29	43.4 (15.9)	45.8	44.2	29	HPL depression scale	Death certificates.
Jonas 2000 (31)	USA	6,095	483	18	NA	NA	NA	16	GDS	Hospital records and death certificates.
Larson 2001 (32)	USA	1,703	95	13	NA	NA	NA	13	CES-D	Self-reports or death certificate.
Ohira 2001 (33)	Japan	901	69	10.3	NA	35	65	10.3	SDS	Death certificate, medical records, or clinical diagnosis.
Ostir 2001 (34)	USA	2,478	340	6	NA	31	69	6	CES-D	Clinical diagnosis or death certificate.
Wassertheil-Smoller 2004 (35)	USA	93,676	751	NA	NA	NA	NA	4.1	CES-D and DIS	Self-reports or proxy-reports.
Gump 2004 (36)	USA	11,216	167	18.4	46	100	0	18.4	CES-D	Death certificates.
Kamphuis 2006 (37)	Finland, Italy, Netherlands	799	66	10	80	100	0	7.4	SDS	Death certificates.
Arbelaez 2007 (38)	USA	5,525	607	11	42	42	58	11	CES-D	Medical records and death certificates.
Salaycik 2007 (39)	USA	4,120	228	NA	NA	NA	NA	8	CES-D	Clinical diagnosis.
Bos 2008 (40)	Netherlands	4,424	291	5.8	72	40	60	5.6	CES-D	Self-reports, clinical diagnosis, computed tomographic scan, or hospital records.
Liebetrau 2008 (41)	Sweden	494	56	3	85	30	70	3	DSMMD-III	Hospital discharge register, death certificates, self-reports, and key informants.
Surtees 2008 (42)	UK	20,627	595	8.5	NA	45.5	54.4	8.5	MHI-5	Clinical diagnosis or death certificate.
Wouts 2008 (43)	Netherlands	2,965	176	7.7	71	48	52	7.7	CES-D	Self-reports or general practitioners’ diagnosis.
Nabi 2010 (44)	Finland	23,282	129	7	64	41	59	7	BDI	Hospital discharge register or mortality records.
Wassertheil-Smoller 1996 (45)	USA	4,736	294	5	72	42.9	57	4.5	CES-D	Clinical diagnosis or death certificates.
Simons 1998 (46)	Australia	2,805	401	8.2	69	44	56	7	Clinical Diagnosis	Hospital and death records
Whooley 1998 (47)	USA	7,518	473	6	72	0	100	NA	GDS	Death certificates and hospital records
May 2002 (48)	Wales	2,172	130	14	56.8	100	0	NA	30-item GHQ	Death certificates and clinical diagnosis
Yasuda 2002 (49)	Japan	980	26	7.5	72	38.8	61.1	7.5	30-item GHQ	Death certificates
Sturmer 2006 (50)	Germany	4,267	62	8.5	53.4	48.2	51.5	NA	Clinical diagnosis	Death certificates
Kawamura 2007 (51)	Japan	920	158	15	NA	40.1	59.8	15	DSMMD-III	Death certificates and clinical diagnosis
Lee 2008 (52)	Taiwan	4,962	98	5	NA	44	56	5	DSMMD-III	Medical records
Whooley 2008 (53)	USA	4,876	341	4.8	67	80	20	NA	9-item PHQ	Hospital records and death certificates
Peters 2010 (54)	UK	2,656	97	2.1	NA	39	59	6	GDS	Medical records

NA, Not Available; CES-D, Center for Epidemiologic Studies Depression Scale; MHI-5, The Mental Health Inventory-5; DSM-IV, Diagnostic and Statistical Manual of Mental Disorders fourth edition; EDS, Euro-Depression Scale; GDS, The Geriatric Depression Scale; HPL, Human Population Laboratory Depression Scale; SDS, The Zung Self-Rating Depression Scale; DSMMD-III, The Diagnostic and Statistical Manual of Mental Disorders Third Edition; BDI, Beck’s Depression Inventory; 30-item GHQ, 30-item General Health Questionnaire; PHQ, The Patient Health Questionnaire; ICD, International Statistical Classification of Diseases and Related Health Problems.

**Figure 2:**
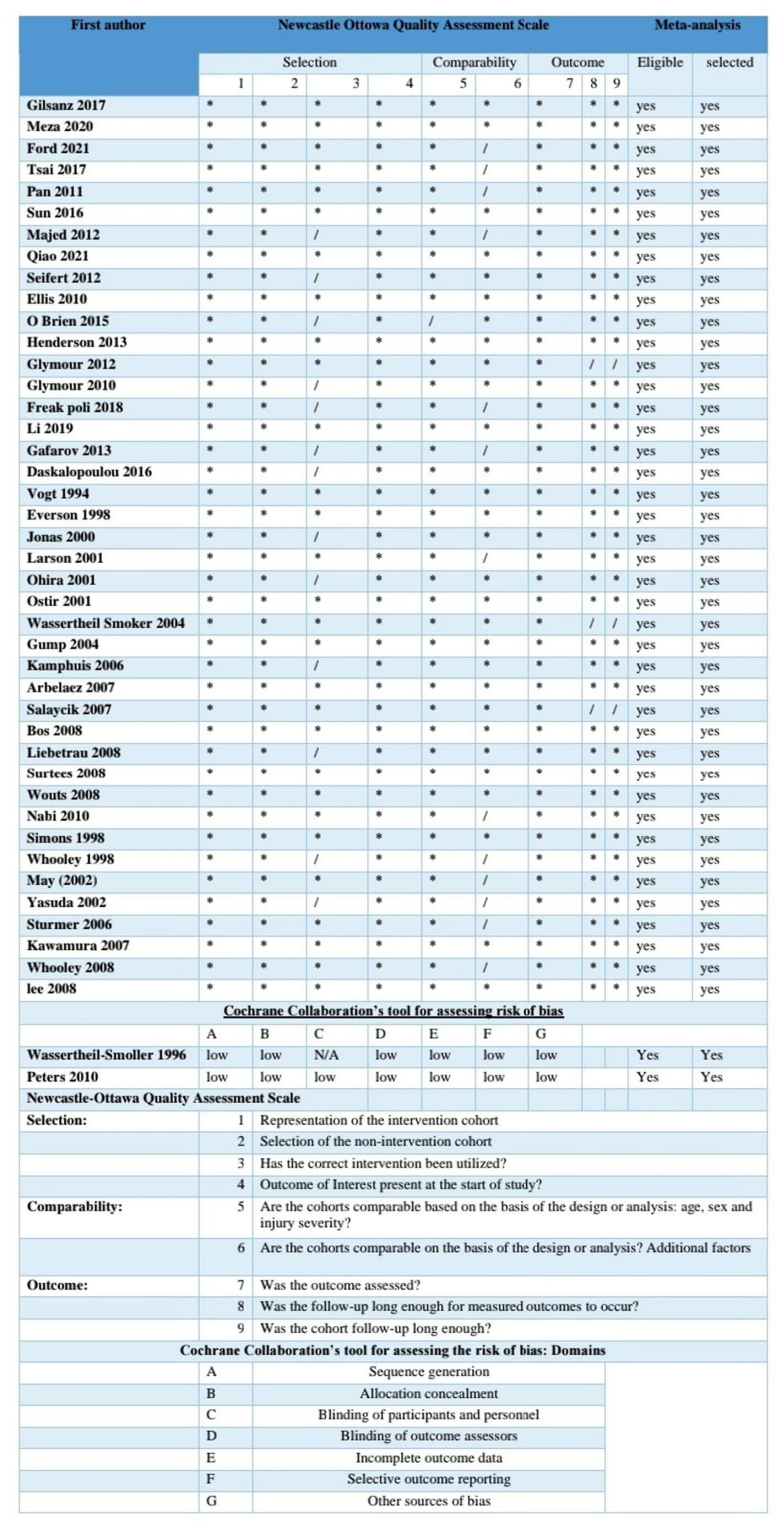
Assessment of risk of bias by Newcastle–Ottawa scale and Cochrane risk of bias tool.

The selected studies were conducted in different regions, primarily in the United States and European countries, with additional studies conducted in Japan, Australia, Taiwan, and as part of an international collaboration. The sample sizes of the studies ranged from 401 to 93,676 participants. The total number of participants and cases were 2,379,274 and 148,389, respectively. The mean follow-up duration was 9.35 ± 5.06 years, and the mean age was 64.01 ± 11.35 years. The subsequent assessment durations varied from 2 to 29 years, allowing for the examination of long-term effects. All the included studies were of high methodological quality, as shown in the risk of bias chart, assessed by the Newcastle–Ottawa Scale and Cochrane risk of bias tools (Figure 2).

### Depression and risk of stroke

**Figure 3:**
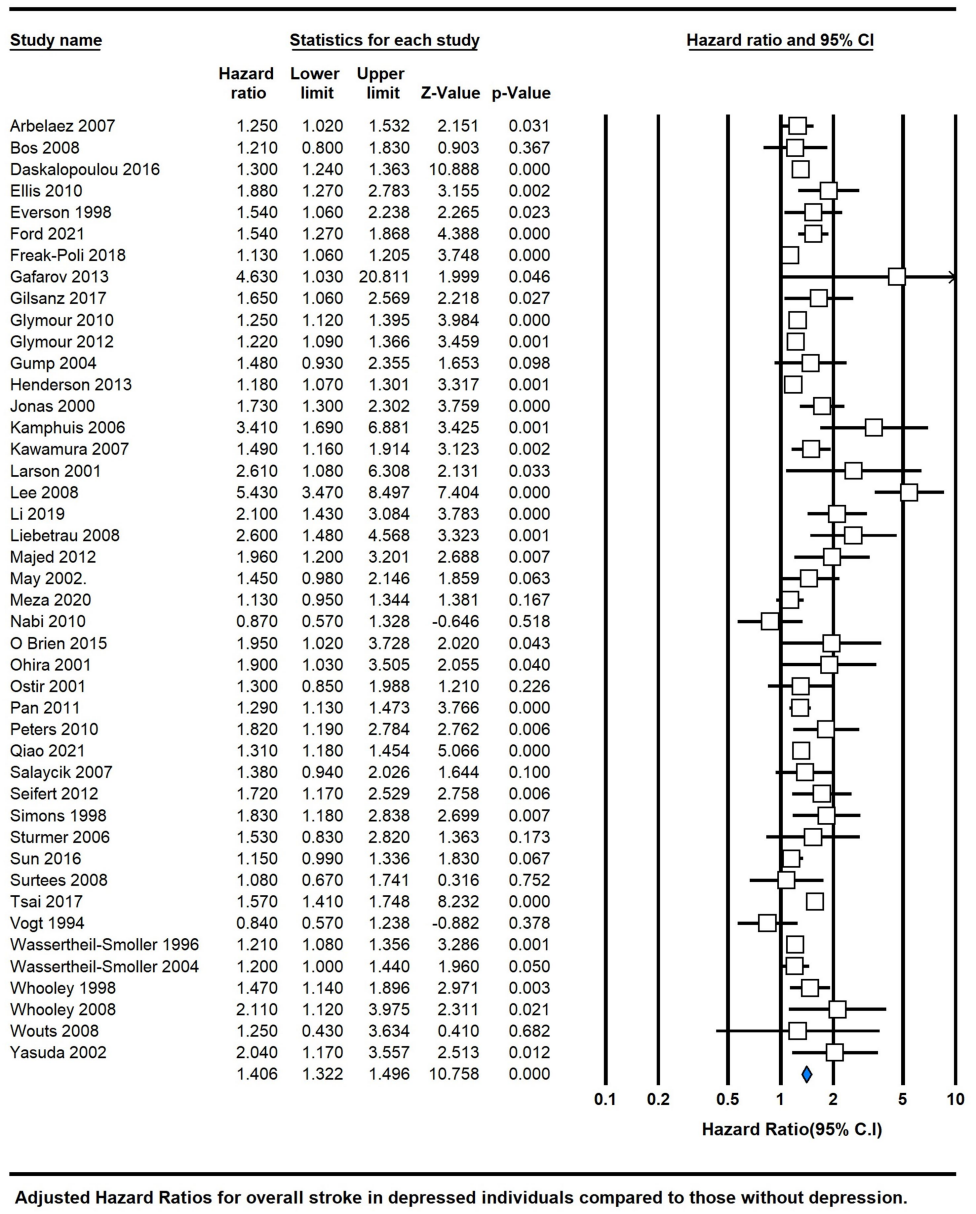
Forest plot of hazard ratios of total stroke for depressed participants compared with non-depressed participants.

The meta-analysis of depression and the risk of stroke included a total of 44 studies (Figure 3). The pooled analysis revealed a significant association between depression and the risk of stroke, with an overall HR of 1.41 (95% CI 1.32, 1.50; *P* < 0.00001), indicating a moderate positive effect size. However, the significant heterogeneity among the studies (I^2 = 71%) suggested substantial variability in the effect estimates.

### Sensitivity analysis

To improve the accuracy and reliability of our research findings, we conducted sensitivity analyses using the leave-one-out method. This approach allowed us to identify the specific studies that contributed to the heterogeneity and assess their impact on our results. By using this technique, we aimed to enhance the precision of our findings and ensure more dependable outcomes (Supplementary File S1). The HR between stroke and depression was 1.35 (95% CI 1.28, 1.43; *p* < 0.00001) after sensitivity analysis.

### Subgroup analysis

**Figure 4:**
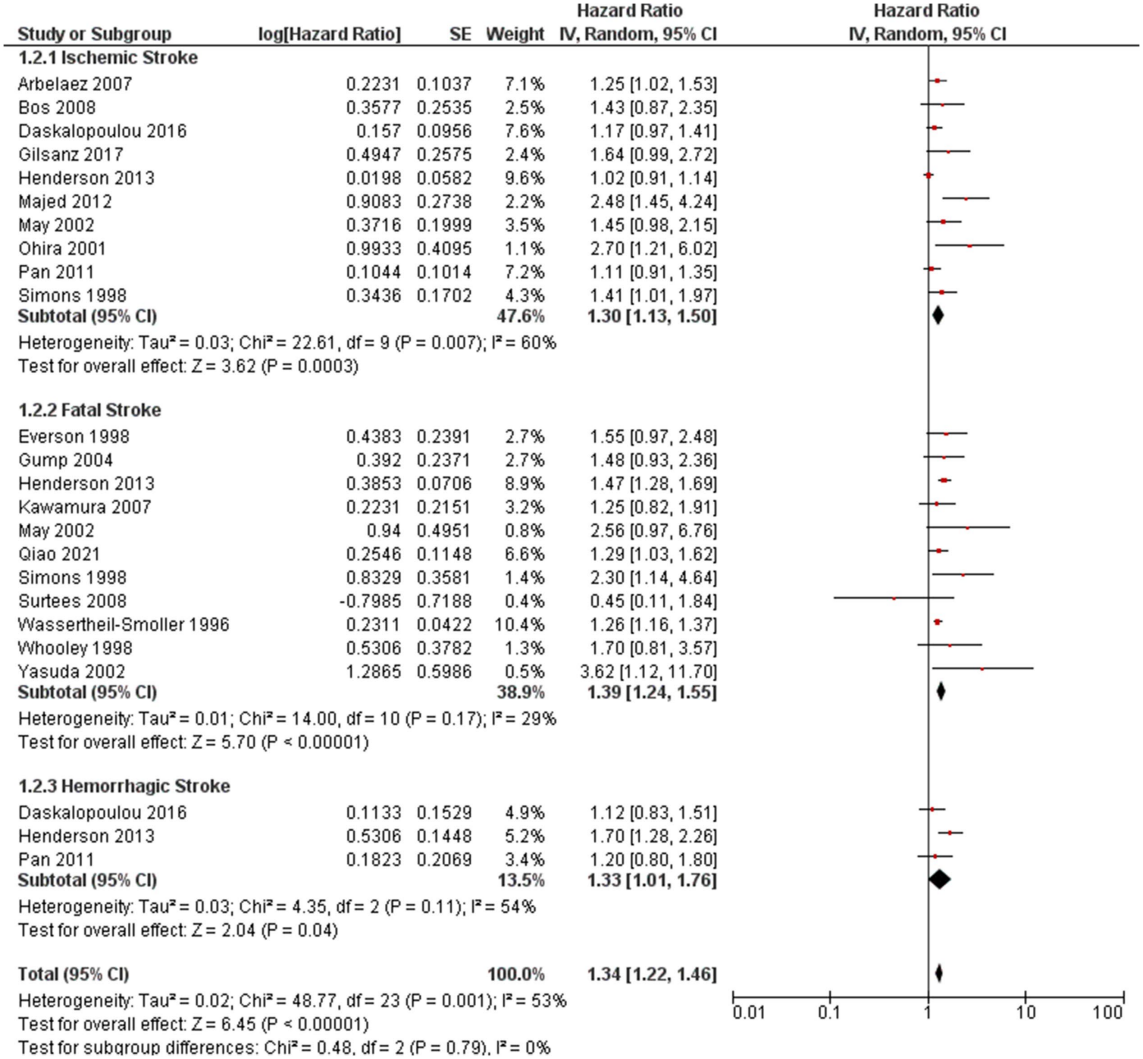
Forest plot of subgroup analysis by type of stroke.

Subgroup analyses were conducted to explore the association between depression and ischemic stroke, between depression and fatal stroke, and between depression and hemorrhagic stroke (Figure 4). There was no significant difference in the effects of subgroups. The HR between depression and ischemic stroke was 1.30 (95% CI 1.13, 1.50; *P* = 0.007), between depression and fatal stroke was 1.39(95% CI 1.24, 1.55; *P* < 0.000001), and between depression and hemorrhagic stroke was 1.33(95% CI 1.01, 1.76; *P* = 0.04).

**Figure 5:**
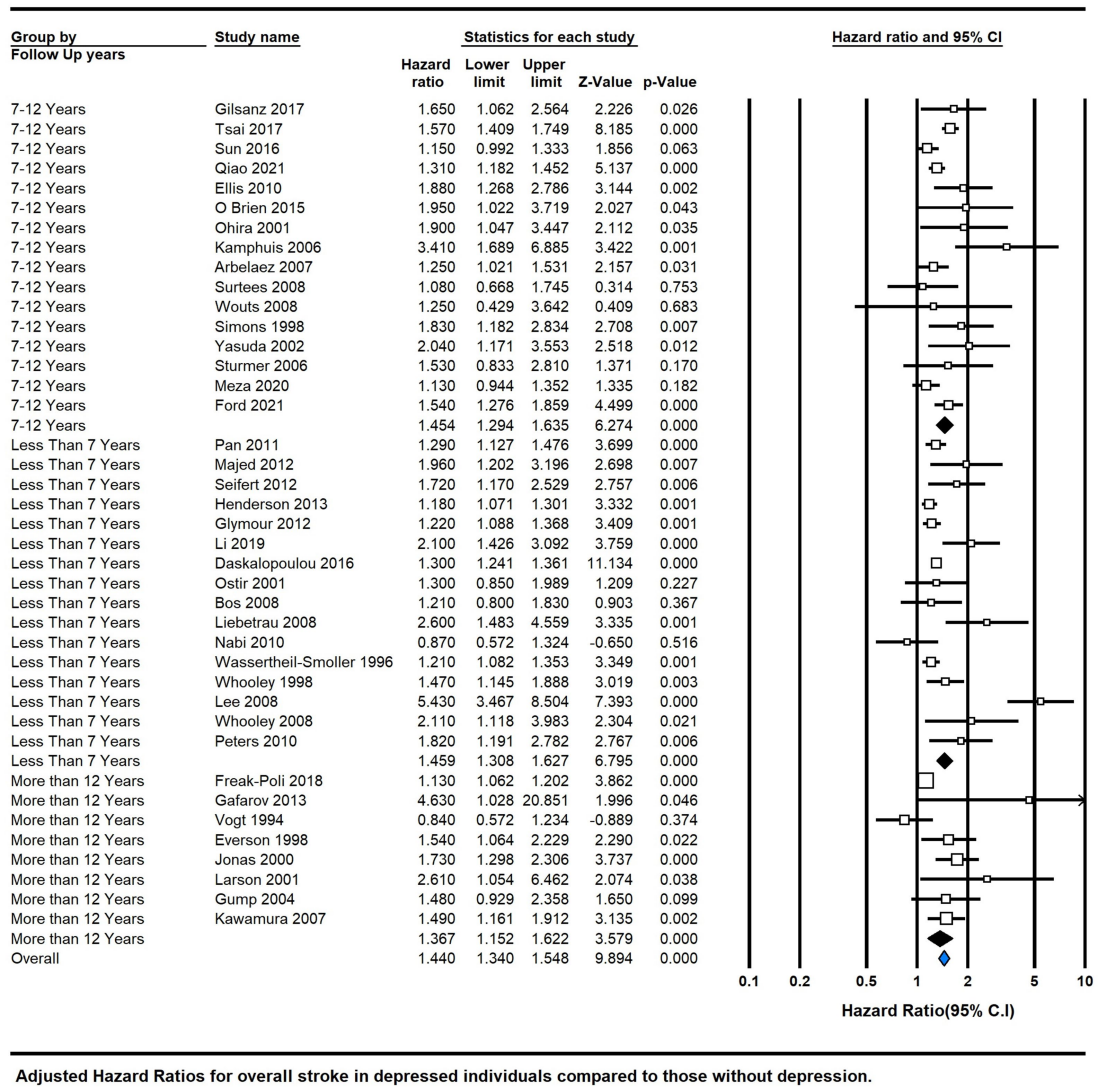
Subgroup analysis by means of follow-up duration.

The analysis further stratified by follow-up duration: less than 7 years (HR = 1.459, 95% CI 1.308–1.627, *p* = 0.000, *I*^2^ = 78.95), 7–12 years (HR = 1.382, 95% CI 1.310–1.459, *P* = 0.000, *I*^2^ = 57.78), and more than 12 years (HR = 1.178, 95% CI 1.113–1.247, *P* = 0.000, *I*^2^ = 70.899). These findings underline the significance of depression in increasing the risk of diverse stroke types, with the association strength varying based on follow-up duration (Figure 5).

### Use of antidepression medication and stroke

**Figure 6:**
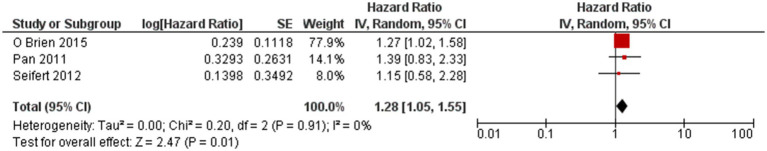
Forest plot of hazard ratios between the use of antidepressants and stroke.

We explored the association between the use of antidepressant medication and stroke. Three studies reported the association, and the overall HR was 1.28 (95% CI 1.05, 1.55; *P* = 0.01). There was a positive association between the use of antidepressant medication and stroke (Figure 6).

### Analysis of publication bias

The analysis of the funnel plot (Supplementary File S1) revealed a possible indication of publication bias in the data, and the Eggers test was significant (intercept 1.386; *p* = 0.00018). Despite the potential presence of publication bias, a trim-and-fill sensitivity analysis was performed, with 13 studies trimmed to assess the robustness of the results. In this case, the results of the trim-and-fill sensitivity analysis showed minimal influence on the findings. Only a slight attenuation, or weakening, of the pooled HR was observed, suggesting that the potential missing studies were unlikely to significantly alter the overall conclusion of the analysis.

## Discussion

Our comprehensive meta-analysis, comprising a total of 44 studies, has uncovered a significant association between depression and the risk of stroke (Figure 3). The combined analysis demonstrated an overall HR of 1.41 (95% CI 1.32, 1.50; *p* < 0.00001), indicating a moderately positive effect size. These findings are in line with a previous meta-analysis published in 2011 (4), which reported adjusted HRs of 1.45 (95% CI, 1.29–1.63) for overall stroke and 1.55 (95% CI, 1.25–1.93) for fatal stroke.

Furthermore, our current meta-analysis provides robust evidence supporting the association between depression and the risks of total stroke, fatal stroke, and ischemic stroke. These results are consistent with the INTERSTROKE study (55), a large case–control study. Subgroup analyses were conducted to explore the relationship between depression and different stroke subtypes, namely ischemic stroke, fatal stroke, and hemorrhagic stroke (Figure 4). Interestingly, no significant differences in the effects were observed among these subgroups, suggesting that the association between depression and stroke may not vary substantially based on the stroke subtype.

It is important to note that three studies (33, 38, 40) reported findings on ischemic stroke, with two studies (33, 38) indicating a significantly increased risk, while one study (33) reported no association with hemorrhagic stroke.

Our analysis revealed an elevated risk of stroke associated with the use of antidepressant medication, with an overall HR of 1.28 (95% CI 1.05, 1.55; *p* = 0.01). These findings are consistent with previous observational studies (56, 57), suggesting a possible link between antidepressant use and stroke, which included selective serotonin reuptake inhibitor (SSRI) and tricyclic antidepressants (TCA), with the risk of ischemic, hemorrhagic, and fatal stroke being higher in SSRIs. However, caution should be exercised when interpreting these findings due to potential confounding factors, such as the severity of depression.

Moreover, age and cardiac disease status were identified as potential modifiers in the association between depression and stroke. In one study (39), depression was significantly associated with an increased risk of stroke only in individuals aged 65 years or younger. Additionally, potential mechanisms underlying the association between depression and stroke were explored. MRI scans have revealed that deep white matter lesions are more frequently observed in older individuals with depression (senile depression) compared to those without depression (58, 59). Furthermore, patients who experience depression at a younger age (presenile-onset depression) exhibit a higher incidence of silent cerebral infarctions, regardless of the specific subtypes of depression (60).

Other established susceptibility factors for stroke, including age, BMI, hypertension, diabetes, smoking, education, and history of cardiovascular events, were thoroughly considered. Notably, cardiac disease serves as a significant contributor to stroke, and depression in patients suffering from cardiovascular disorders may exacerbate the underlying atherosclerotic disease, potentially leading to stroke (43). Detrimental lifestyle elements, such as smoking (61) and reduced exercise levels (62), which themselves increase the risk of depression, were also found to be associated with depression.

Furthermore, inflammatory markers such as CRP, IL-1, and IL-6 have shown positive associations with depression in both clinical and community samples (6). The magnitude of the association between depression and stroke observed in our study is comparable to the associations between smoking and stroke (63).

The potential occurrence of reverse causality should be carefully considered when examining the association between depression and stroke, since some studies have suggested that a history of stroke might enhance the likelihood of developing depression (64). It is possible that certain studies may have overlooked prior stroke incidents that could have led to both depression and subsequent stroke episodes. Furthermore, apart from the studies included in our meta-analysis, several other studies that did not meet our inclusion criteria have also reported a positive association between depression and stroke. For example, Simonsick et al. (65) observed significantly higher rates of stroke incidence in subgroups of older adults with hypertension exhibiting high depressive symptoms compared to non-depressed individuals. Similarly, Nilsson and Kessing et al. (66) found increased future stroke risk among hospitalized patients with severe depression in comparison to patients with osteoarthritis.

Moreover, it is important to note that further research is necessary to investigate the association between depression and stroke in non-Caucasian populations. One study revealed a relatively high prevalence of elevated depressive symptoms among the Caucasian population, the Hispanic population, and the Black population, with Hispanics having the highest occurrence (10). This finding highlights the need for comprehensive studies encompassing diverse populations to gain a better understanding of the relationship between depression and incident stroke.

### Strength and limitations

Several limitations should be acknowledged in our meta-analysis. First, significant heterogeneity was observed across the included studies, likely stemming from variations in study designs, sample sizes, measures of depression and stroke, analysis approaches, and participant characteristics. Although moderate to high heterogeneity persisted in several subgroups, the pooled HRs consistently indicated positive associations. The primary contributors to the heterogeneity were three specific studies (15, 25, 52) that had distinct participant criteria, such as age restrictions and inclusion of rheumatoid arthritis patients. A sensitivity analysis was conducted for these studies, and the forest plot can be found in Supplementary File S1. Furthermore, the analysis of the funnel plot indicated the potential presence of publication bias. However, when conducting the trim-and-fill sensitivity analysis, the results were minimally affected, with only a slight attenuation observed in the pooled HR. One notable strength of our study lies in its extensive inclusion of 44 studies, surpassing the number of studies incorporated in previous meta-analyses. This comprehensive analysis provides compelling evidence concerning the association between depression and the increased risk of stroke.

Another potential limitation of this study is the absence of a separate analysis based on gender. Due to the lack of available data in the existing literature, we were unable to conduct gender-specific analyses. This omission is significant considering the widely suggested notion of a female preponderance in depression, which is deemed universal and substantial. Previous studies have established this association (67, 68). Hence, it is imperative for future research endeavors to delve deeper into the relationship between depression, gender, and the risk of stroke, as understanding these dynamics could yield valuable insights into preventive strategies and tailored interventions.

## Conclusion

In summary, our exhaustive meta-analysis strongly establishes the association between depression and an increased risk of stroke, validating earlier findings and indicating potential involvement across various stroke subtypes. Furthermore, our study highlights a significant association between heightened stroke risk and the use of antidepressant medication. Moving forward, further research is essential to unravel the underlying mechanisms of this connection and deepening our understanding of the intricate interrelationship between depression and stroke. Additionally, investigations into effective interventions for reducing stroke risk in individuals with depression are crucial while simultaneously scrutinizing the role of antidepressants in this context, encompassing their impact on stroke risk and associated mechanisms. This comprehensive approach aims to provide a holistic viewpoint on this complex issue.

## Data availability statement

The original contributions presented in the study are included in the article/Supplementary material, further inquiries can be directed to the corresponding author/s.

## Author contributions

FA: Writing – review & editing, Conceptualization. MM: Writing – review & editing, Supervision, Methodology, Formal analysis. MSh: Writing – original draft, Investigation, Conceptualization. AH: Writing – original draft, Data curation. AM: Writing – original draft. AN: Writing – original draft. BS: Writing – original draft. FK: Writing – original draft. MS: Writing – original draft. JI: Writing – original draft.
