# Supplementary material for: Association between depression and stroke risk in adults: a systematic review and meta-analysis
Source: Front Neurol. 2024 Apr 25;15:1331300. doi: 10.3389/fneur.2024.1331300 (PMC11079212; doi:10.3389/fneur.2024.1331300)
Supplement: Supplementary file 1 [file Image_1.pdf]

Supplementary file 1

Supplementary Figure 1a: Forest plot showing sensitivity analysis of Depression and Stroke

Study or Subgroup	log[Hazard Ratio]	SE	Weight	Hazard Ratio IV, Random, 95% CI
Arbelaez 2007	0.2231	0.1037	3.7%	1.25 [1.02, 1.53]
Bos 2008	0.1906	0.2111	1.4%	1.21 [0.80, 1.83]
Daskalopoulou 2016	0.2624	0.0241	7.5%	1.30 [1.24, 1.36]
Ellis 2010	0.6313	0.2001	1.5%	1.88 [1.27, 2.78]
Everson 1998	0.4318	0.1906	1.6%	1.54 [1.06, 2.24]
Ford 2021	0.4318	0.0984	3.9%	1.54 [1.27, 1.87]
Freak-Poli 2018	0.1222	0.0326	0.0%	1.13 [1.06, 1.20]
Gafarov 2013	1.5326	0.7668	0.1%	4.63 [1.03, 20.81]
Gilsanz 2017	0.5008	0.2258	1.2%	1.65 [1.06, 2.57]
Glymour 2010	0.2231	0.056	6.0%	1.25 [1.12, 1.39]
Glymour 2012	0.1989	0.0575	5.9%	1.22 [1.09, 1.37]
Gump 2004	0.392	0.2371	1.1%	1.48 [0.93, 2.36]
Henderson 2013	0.1655	0.0499	6.3%	1.18 [1.07, 1.30]
Jonas 2000	0.5481	0.1458	2.4%	1.73 [1.30, 2.30]
Kamphuis 2006	1.2267	0.3582	0.5%	3.41 [1.69, 6.88]
Kawamura 2007	0.3988	0.1277	2.9%	1.49 [1.16, 1.91]
Larson 2001	0.9594	0.4502	0.4%	2.61 [1.08, 6.31]
Lee 2008	1.6919	0.2285	0.0%	5.43 [3.47, 8.50]
Li 2019	0.7419	0.1961	1.6%	2.10 [1.43, 3.08]
Liebetrau 2008	0.9555	0.2875	0.8%	2.60 [1.48, 4.57]
Majed 2012	0.6729	0.2503	1.0%	1.96 [1.20, 3.20]
May 2002	0.3716	0.1999	1.5%	1.45 [0.98, 2.15]
Meza 2020	0.1222	0.0885	4.3%	1.13 [0.95, 1.34]
Nabi 2010	-0.1393	0.2157	1.3%	0.87 [0.57, 1.33]
O Brien 2015	0.6678	0.3306	0.6%	1.95 [1.02, 3.73]
Ohira 2001	0.6419	0.3124	0.7%	1.90 [1.03, 3.51]
Ostir 2001	0.2624	0.2168	1.3%	1.30 [0.85, 1.99]
Pan 2011	0.2546	0.0676	5.4%	1.29 [1.13, 1.47]
Peters 2010	0.5988	0.2168	1.3%	1.82 [1.19, 2.78]
Qiao 2021	0.27	0.0533	6.1%	1.31 [1.18, 1.45]
Salaycik 2007	0.3221	0.1959	1.6%	1.38 [0.94, 2.03]
Seifert 2012	0.5423	0.1966	1.6%	1.72 [1.17, 2.53]
Simons 1998	0.6043	0.2239	1.3%	1.83 [1.18, 2.84]
Sturmer 2006	0.4253	0.312	0.7%	1.53 [0.83, 2.82]
Sun 2016	0.1398	0.0764	4.9%	1.15 [0.99, 1.34]
Surtees 2008	0.077	0.2436	1.1%	1.08 [0.67, 1.74]
Tsai 2017	0.4511	0.0548	0.0%	1.57 [1.41, 1.75]
Vogt 1994	-0.1744	0.1978	1.5%	0.84 [0.57, 1.24]
Wassertheil-Smoller 1996	0.1906	0.058	5.9%	1.21 [1.08, 1.36]
Wassertheil-Smoller 2004	0.1823	0.093	4.2%	1.20 [1.00, 1.44]
Whooley 1998	0.3853	0.1297	2.9%	1.47 [1.14, 1.90]
Whooley 2008	0.7467	0.3231	0.7%	2.11 [1.12, 3.97]
Wouts 2008	0.2231	0.5445	0.2%	1.25 [0.43, 3.63]
Yasuda 2002	0.7129	0.2837	0.8%	2.04 [1.17, 3.56]
**Total (95% CI)**			**100.0%**	**1.35 [1.28, 1.43]**

Heterogeneity: Tau² = 0.01; Chi² = 78.51, df = 40 (P = 0.0003); I² = 49%
Test for overall effect: Z = 11.08 (P < 0.00001)

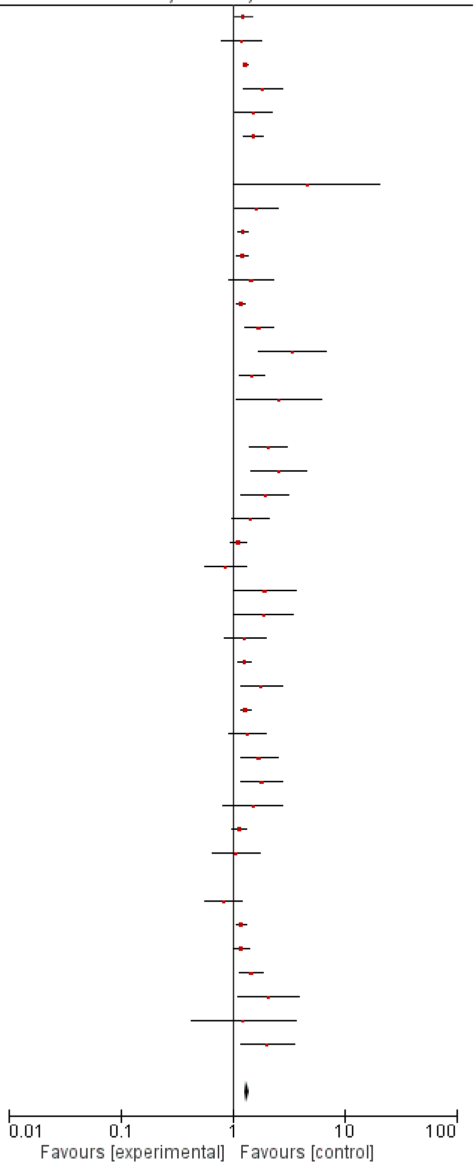

Figure 1 Sensitivity analysis of depression and Stroke

Supplementary Figure 1b: Leave one out analysis

Study name	Statistics with study removed					Hazard ratio (95% CI) with study removed
	Point	Lower limit	Upper limit	Z-Value	p-Value	
Arbelaez 2007	1.413	1.326	1.506	10.645	0.000	
Bos 2008	1.410	1.324	1.502	10.720	0.000	
Daskalopoulou 2016	1.428	1.332	1.532	10.009	0.000	
Ellis 2010	1.398	1.314	1.488	10.553	0.000	
Everson 1998	1.404	1.319	1.495	10.595	0.000	
Ford 2021	1.402	1.316	1.493	10.473	0.000	
Freak-Poli 2018	1.422	1.334	1.515	10.847	0.000	
Gafarov 2013	1.403	1.318	1.492	10.724	0.000	
Gilsanz 2017	1.403	1.318	1.494	10.605	0.000	
Glymour 2010	1.418	1.329	1.513	10.535	0.000	
Glymour 2012	1.419	1.330	1.514	10.588	0.000	
Gump 2004	1.406	1.320	1.497	10.644	0.000	
Henderson 2013	1.421	1.332	1.516	10.640	0.000	
Jonas 2000	1.398	1.313	1.488	10.504	0.000	
Kamphuis 2006	1.395	1.312	1.483	10.664	0.000	
Kawamura 2007	1.404	1.318	1.496	10.534	0.000	
Larson 2001	1.402	1.317	1.491	10.674	0.000	
Lee 2008	1.360	1.288	1.436	11.072	0.000	
Li 2019	1.394	1.310	1.483	10.541	0.000	
Liebetrau 2008	1.395	1.312	1.484	10.613	0.000	
Majed 2012	1.400	1.315	1.490	10.594	0.000	
May 2002.	1.406	1.320	1.497	10.626	0.000	
Meza 2020	1.419	1.331	1.512	10.786	0.000	
Nabi 2010	1.416	1.330	1.507	10.947	0.000	
O Brien 2015	1.402	1.318	1.493	10.643	0.000	
Ohira 2001	1.402	1.318	1.493	10.637	0.000	
Ostir 2001	1.409	1.323	1.500	10.685	0.000	
Pan 2011	1.415	1.326	1.509	10.534	0.000	
Peters 2010	1.400	1.315	1.490	10.575	0.000	
Qiao 2021	1.415	1.326	1.511	10.451	0.000	
Salaycik 2007	1.407	1.322	1.499	10.646	0.000	
Seifert 2012	1.401	1.316	1.491	10.567	0.000	
Simons 1998	1.400	1.316	1.490	10.581	0.000	
Sturmer 2006	1.406	1.320	1.496	10.670	0.000	
Sun 2016	1.419	1.331	1.513	10.750	0.000	
Surtees 2008	1.411	1.326	1.503	10.779	0.000	
Tsai 2017	1.396	1.311	1.485	10.472	0.000	
Vogt 1994	1.417	1.332	1.508	11.012	0.000	
Wassertheil-Smoller 1996	1.419	1.330	1.514	10.608	0.000	
Wassertheil-Smoller 2004	1.416	1.329	1.509	10.687	0.000	
Whooley 1998	1.405	1.319	1.497	10.544	0.000	
Whooley 2008	1.401	1.317	1.491	10.634	0.000	
Wouts 2008	1.407	1.322	1.498	10.731	0.000	
Yasuda 2002	1.400	1.316	1.490	10.614	0.000	
	1.406	1.322	1.496	10.758	0.000	

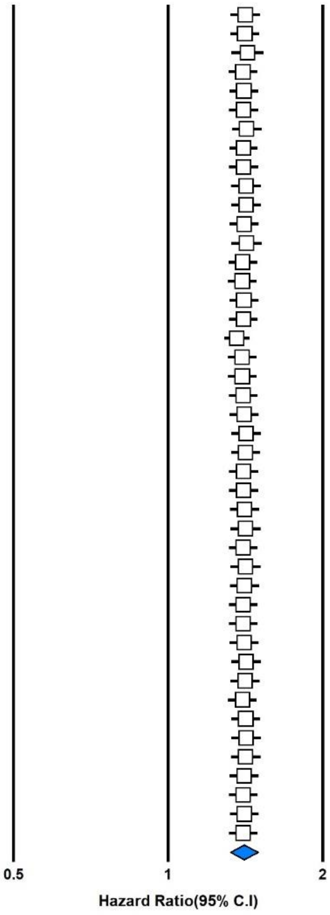

Adjusted Hazard Ratios for overall stroke in depressed individuals compared to those without depression.

Supplementary Figure 1c: Funnel plot for publication bias

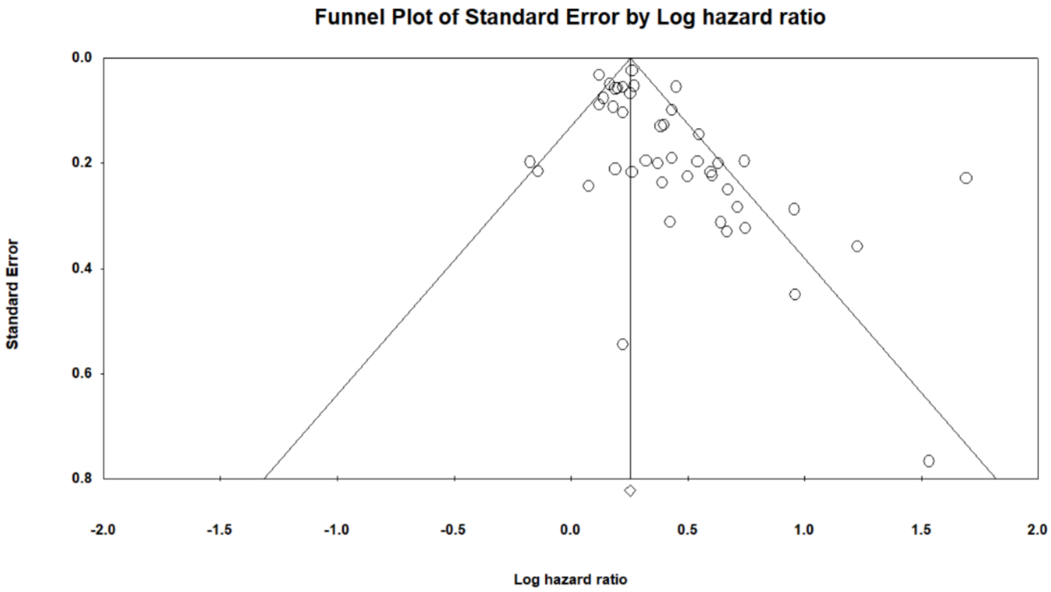

Eggers Regression test for Publication bias

Egger's regression intercept

Intercept	1.38695
Standard error	0.35721
95% lower limit (2-tailed)	0.66607
95% upper limit (2-tailed)	2.10784
t-value	3.88270
df	42.00000
P-value (1-tailed)	0.00018
P-value (2-tailed)	0.00036

Trim and Fill Method:

Duval and Tweedie's trim and fill

		Fixed Effects			Random Effects			Q Value
	Studies Trimmed	Point Estimate	Lower Limit	Upper Limit	Point Estimate	Lower Limit	Upper Limit	
**Observed values**		1.28817	1.25630	1.32084	1.40624	1.32155	1.49635	147.52937
**Adjusted values**	13	1.26719	1.23628	1.29888	1.30096	1.21681	1.39094	227.21756
